# Supplementary material for: The hydrophobic effect characterises the thermodynamic signature of amyloid fibril growth
Source: PLoS Comput Biol. 2020 May 4;16(5):e1007767. doi: 10.1371/journal.pcbi.1007767 (PMC7282669; doi:10.1371/journal.pcbi.1007767)
Supplement: S1 Methods — (PDF) [file pcbi.1007767.s001.pdf]

Simulation methods

Definition of states

We observe four distinct states, which for the analysis are defined based on the number of external contacts for the two free peptides; external contacts (C_{ext}) are here defined as the interchain contacts between either the free peptides or the free peptides and the seed. Note that there are a further 70 external contacts present in the seed fibril that cannot be changed during the simulations.

The monomeric, fibrillar and amorphous states are then defined as:

$$monomer = \begin{cases} 1 \; if \; C_{ext} = 0 \\ 0 \; otherwise \end{cases}$$

$$amorphous = \begin{cases} 1 \; if \; 11 < \; C_{ext} \leq 14 \\ \quad 0 \; otherwise \end{cases}$$

$$fibril = \begin{cases} 1 \; if \; C_{ext} \geq 16 \\ \; 0 \; otherwise \end{cases}$$

$$fully\ aggregated = \begin{cases} 1 \; if \; C_{ext} = 21 \\ \; 0 \; otherwise \end{cases}$$

Enthalpy sampling

For the simulation we calculate the enthalpy difference between the ensemble of the fully aggregated state ($\mathrm{A}: \mathrm{C_{ext}} = 21$) and the monomeric state ($\mathrm{M}: \mathrm{C_{ext}} = 0$); in the latter case only the seed peptides still make contacts. The enthalpy difference $\Delta\mathrm{E}$ is then calculated as $\Delta\mathrm{E} = \langle \mathrm{E} \rangle_\mathrm{A} - \langle \mathrm{E} \rangle_\mathrm{M}$.

Hydrogen bonds

The term describing hydrogen bonds can be written as $\mathrm{E_{hb}} = \sum \epsilon_\mathrm{hb} \mathrm{H_{i,j}} \cdot \mathrm{C_{i,j}}$, where ϵ_hb represents the potential energy per hydrogen bond. $\mathrm{H_{i,j}} = 1$ indicates that a hydrogen bond is present, and $\mathrm{H_{i,j}} = 0$ indicates that no hydrogen bond is present. In our model, the water interactions are implicit, so ϵ_hb indicates the difference between a hydrogen bond of an amino acid with the solvent and a hydrogen bond in bulk water. Hydrogen bonds between amino acids and the solvent are typically stronger than the hydrogen bonds in a bulk solvent. We investigate the cases $\epsilon_\mathrm{hb} \in \{0.25, 0.5, 0.75, 1.0\}$ with $\epsilon_\mathrm{hb} = 0.5$ as default.

Entropically favourable β-sheets

We also investigated the effect of an entropic bonus for β-sheets. The high stability of β-sheets at elevated temperatures suggests that β-sheets may have a higher entropy than other secondary structure elements. Moreover, amino acids with a high propensity for β-strand formation tend to be β-branched [1], suggesting that the β-strand states will be entropically more favourable. Core regions of amyloid forming proteins tend to have a high β-strand forming propensity [2], to model this propensity we allow an entropic term, N_β, to be set for the β-strand state modelling degeneracy of the state.

This allows us to investigate the effect of a local entropic 'bonus' a residue receives for being in a β-sheet. Unless otherwise stated $\mathrm{N}_\beta = 1$, giving no bias.

Sampling analysis

We used the umbrella sampling method to sample the conformational space [3]. As order parameter, we used the number of external contacts, C_{ext}. We use a quadratic biasing potential to define E_{umbr}:

$$E_{umbr} = \mathcal{H} + k(C_{ext} - C_{ext,0})^2$$

Where k is the spring constant, $\mathcal{H}$ the Hamiltonian defined in eqn. 10 (see Materials and Methods), and $C_{ext,0}$ the value towards which the simulation is biased. In our simulations, $k = 2$ and $C_{ext,0} \in \{0,5,10,15,20,25\}$.

References

1. Chou KC, Némethy G, Scheraga HA. Role of interchain interactions in the stabilization of the right-handed twist of β-sheets. Journal of molecular biology. 1983;168(2):389–407
2. Trovato A, Chiti F, Maritan A, Seno F. Insight into the structure of amyloid fibrils from the analysis of globular proteins. PLoS computational biology. 2006;2(12):e170
3. Grossfield A. WHAM: the weighted histogram analysis method; 2003
