# Supplementary material for: The hydrophobic effect characterises the thermodynamic signature of amyloid fibril growth
Source: PLoS Comput Biol. 2020 May 4;16(5):e1007767. doi: 10.1371/journal.pcbi.1007767 (PMC7282669; doi:10.1371/journal.pcbi.1007767)
Supplement: S2 Methods — (PDF) [file pcbi.1007767.s002.pdf]

# Experimental methods

## Enthalpy of GNNQQNY crystal growth

GNNQQNY peptide was bought from Bachem (Basel, Switzerland) as TFA salt and used without further purification. The peptide was dissolved in hot water at a concentration of 2-3 mM and left to cool down. Crystal formation occurred spontaneously after some time, but could be strongly accelerated by probe sonication. The stock solutions were kept for several days at room temperature.

A concentrated, super-critical solution of GNNQQNY peptide was found not to remain metastable for a sufficient amount of time to be used in seeded ITC experiments, such as the ones described above for amyloid fibrils. Therefore, the experiments to determine the heat of formation of GNNQQNY crystals as a function of temperature were designed as crystal dissolution experiments, rather than crystal growth experiments. Before each calorimetry experiments, the suspensions were re-sonicated for 2 min to homogenise them. Different dilutions of the freshly sonicated stock solutions were loaded into the syringe of a VP-ITC and the cell filled with pure water. Then large injections of up to 100 µl were performed. The injection of crystals into water always yielded strongly endothermic signatures. It was noticed that identical injections yielded strongly differing peak integrals. This was found to be due to the sedimentation of the crystals inside the injection syringe, which leads to the fact that injections of equal volumes do not correspond to equal quantities of injected peptide crystals.

Therefore, in order to determine the enthalpy of crystal dissolution, the total integrated heat of all injections was determined and this value was correlated with the final peptide concentration in the cell after the experiment, which was measured by UV absorption measurements at 280 nm (molar extinction coefficient 1280 $\mathrm{M^{-1}cm^{-1}}$).

When a suspension of crystals is injected into water, this corresponds to the injection of the crystals themselves, plus the injection of monomer at the critical concentration. In order to be able to determine the net heat of crystal dissolution, the obtained experimental values need to be corrected for the values of the heat of dilution of the present monomer. In order to quantify this correction, samples of crystal suspensions were heated to 60°C for 30 min, which led to the dissolution of the crystals. The resulting supersaturated solution was loaded into the needle of the ITC calorimeter and the same pattern of injections was performed as for the crystals. In some cases, the re-crystallisation started inside the needle, which lead to a drift in the baseline. However, in all cases, the injection of "melted" crystals led to an exothermic signal. After these control experiments, the concentration of the peptide inside the cell was measured again.

It was found that at 20°C, the heat of dilution of monomer was -187.4 µcal for a final concentration of 273 µM. The critical concentration of GNNQQNY was found to be ~110 µM at 20°C. Assuming that the heat of dilution scales linearly with final concentration, we subtracted -13 µJ from each experimental data point at 20°C.

Furthermore, it was found that at 30°C, the heat of dilution of monomer was -213.1 µcal for an injected concentration of 305 µM, which yielded a final concentration of 53 µM. The critical concentration of GNNQQNY was found to be ~154 µM at 30°C. We therefore subtracted -108 µJ from each experimental data point at 30°C.

The measured heats of crystal dissolution are then plotted against the concentration in the ITC cell at the end of the experiment. A linear fit forced to go through 0/0 yields the molar heat of crystal dissolution. We find 4.2 cal/mol and 10.3 cal/mol at 20°C and 30°C, respectively, yielding a value of 0.61 cal/(mol K) or 2.6 J/(mol K) as value for the molar heat capacity of crystal dissolution. We fitted Equation 11 through the data points to estimate γ. Note that in this case, we only have data at two temperatures and therefore we cannot estimate the accuracy of the fit. However, this data and analysis does provide a good idea about the magnitude of the enthalpy and heat capacity of GNNQQNY assembly into crystals.

## Chemical denaturation experiments

For α-lactalbumin (S6A Fig, 200 µl of 377 µM fibrils was mixed with 1000 µl solution mixture of variable ratios of (1) 10 mM HCl + 100 mM NaCl and (2) 6M GndSCN + 10 mM HCl + 100 mM NaCl, in order to obtain a series of increasing denaturant concentration, ranging from 1.2 M to 5 M. The samples were incubated for 10 days at room temperature and then centrifuged for 1 h at 25°C at 40 krpm. The equilibrium concentration of soluble α-lactalbumin in the supernatant was determined using the Bradford test. The use of Bradford reagent was necessary, as the denaturant GndSCN has a considerable absorbance at 280 nm and therefore interferes with concentration determination through absorbance measurements at 280 nm, as well as with measurements of intrinsic fluorescence (see description for glucagon below). The samples were diluted 1:25 into the Bradford reagent and the values of absorbance at 595 nm were compared with a standard curve.

In the case of glucagon (S6B Fig), the protocol follows closely the one from Vettore et al. [1], except that the fibrils were depolymerised with GndHCl. 32 µl of 300 µM fibril solution (in 10 mM HCl and 30 mM NaCl) was mixed with 128 µl solution mixture of variable ratios of (1) 10 mM HCl + 30 mM NaCl and (2) 8.4 M GndHCl + 10 mM HCl + 30 mM NaCl. The samples were incubated for two days at room temperature. Then the intrinsic fluorescence was measured with a Tecan M1000 multiwell-platereader. The fluorescence emission spectra upon excitation at 280 nm was measured from 300 to 420 nm. The ratio of the fluorescence intensities at 340 nm and 320 nm was normalised for the initial and final plateau values and plotted as a function of the concentration of GndHCl. This measurement had been calibrated against the concentration of soluble peptide, determined by centrifugation and absorbance measurements [1]. The depolymerisation curves (S6 Fig) were fitted with the expression derived from the linear polymerisation model [2,3]. The free energy differences between the soluble and fibrillar states extracted from the fits are -52.5 kJ/mol for α-lactalbumin and -51.2 kJ/mol for glucagon. The value of the thermodynamic stability obtained here for glucagon amyloid fibrils is significantly higher than the one we recently reported for fibrils made under different solution conditions [1]. This difference can be explained by the formation of different fibril strains under different conditions [4] and the fact that in the present study we used an ionic denaturant. If GndHCl is used as a denaturant, the electrostatic repulsion between the monomer units is fully screened, leading to increased absolute fibril stability.

We also performed chemical depolymerization experiments of α-synuclein amyloid fibrils. α-syn does not naturally contain Trp residues, therefore we created a variant where the phenylalanine (Phe) in position 94 has been exchanged with a Trp, α-synuclein F94W. α-syn F94W presents a maximum fluorescence emission peak at 340 nm in the monomeric form, whereas the emission maximum is at 330 nm when the monomer is incorporated in a fibril. In this case, we performed the denaturation experiments with urea, in order to be able to probe the importance of electrostatic effects in the

stability of α-synuclein amyloid fibrils [1]. If an ionic denaturant is used, electrostatic interactions are mostly screened by the high denaturant concentrations used.

F94W α-syn fibrils were prepared in PBS and 0.02% $NaN_3$ and they were probe-sonicated with a MS-72 tip (BANDELIN electronic) before denaturation. Urea stock solutions were prepared at 8 M by dissolving 12 g urea (SigmaAldrich) in ca. 20 ml of buffer (PBS or PB) to reach a final volume of 25 ml. The samples with a fibril concentration of 60 µM (monomer equivalents) and different urea concentrations were left 6 days at approximately 28°C to reach equilibrium as assessed by fluorescence measurements immediately after the sample preparation and one, three and six days later. For the cold destabilisation experiments, the samples were stored at 4°C for up to 28 days and the intrinsic fluorescence spectra were regularly measured. The fluorescence emission spectra were fitted to a Gaussian curve and the ratio of the fluorescence intensities at 340 and 330 nm from the fits was plotted against the urea concentration, reducing the noise level compared to a use of the raw fluorescence intensity values. In Fig. S8 we show that the fluorescence intensity ratio of monomeric α-syn is not influenced by the used salt or maximal urea concentrations and that therefore the observed time evolution of fluorescence intensity ratio can be attributed to the equilibration of the fibrils. The resulting denaturation curves were normalised between 0 and 1, representing the monomer concentration of the samples, and fitted with the isodesmic linear polymerisation model, see above.

The equilibration of the samples at 4°C was followed by monitoring the evolution of the fitted free energy values (S9 Fig). The equilibrium free energy values for F94W α-syn fibrils in PB are -36.1 kJ/mol at 28°C and -24.1 kJ/mol at 4°C and in PBS the free energies amount to -37.4 kJ/mol at 28°C and -28.9 kJ/mol at 4°C. These values of the free energy are similar to the value reported previously for α-syn fibrils (-33 kJ/mol [3]).

In addition to the cold denaturation experiments with the F94W variant of α-synuclein, using intrinsic fluorescence, we also performed some control experiments with the wild type (WT) sequence (S10 Fig). The cold denaturation of WT α-syn fibrils was assessed by Thioflavin-T (ThT) fluorescence and circular dichroism (CD) spectroscopy.

For the ThT experiments, WT α-syn monomers were loaded onto a Superdex 200 Increase 10/300 GL column (GE Healthcare) in 10 mM PB pH 6.5, 0.02% $NaN_3$. The monomers were then converted into fibrils in the presence of a magnetic stirrer under shaking conditions at 700 rpm and at 37°C. The fibrils were centrifuged in a bench top centrifuge 5415R (Eppendorf, Germany) at 16000 g for 30 minutes at 25°C. The pellet was then resuspended in 50 mM Tris-HCl pH 7.5 The urea stock solution was prepared as described above and the samples were added to a low binding and clear-bottom half-area 96 well plate (Corning n° 3881). The cold denaturation of the fibrils was followed under quiescent conditions in a Fluorostar Omega (BMG Labtech,Germany) plate-reader with ThT fluorescence (excitation with a 448-10 nm filter, emission with a 482-10 nm filter, bottom optics) at 7°C (instrument placed in a cold cabinet).

The first fluorescence intensity readings after temperature equilibration were taken to represent the equilibrium fibril mass distribution as a function of urea concentration at room temperature. The final intensity values (time 40 h) were normalized by the initial intensities values (time 0 h) and were taken to correspond to the equilibrium fibril mass distribution as a function of urea concentration at 7°C (S10 Fig).

In addition, we performed CD experiments (S10B Fig) to monitor the time course of fibril dissociation at low temperature (7°C). α-syn fibrils were prepared as described above for the ThT experiments. After 4 days, the sample was centrifuged, as described above, and the supernatant was used to quantify the concentration of monomers in the supernatant by SDS-PAGE. The fibril pellet was resuspended in 50 mM Tris-HCl pH 7.5 in order to obtain a final concentration of circa 20 μM (monomer equivalents), before sonication in an ultrasonic bath for 1 minute. The CD spectra were acquired at 7°C in a J-815 CD spectrometer (Jasco, Japan) in a time course with 4 minutes between measurements. The values at 218 nm and 195 nm were plotted as function of time, as these wavelengths are representative for random coil (α-syn monomers) and β-sheet (α-syn fibrils) structure.

## References


1. Vettore N, Buell A. Thermodynamics of amyloid fibril formation from chemicalde polymerization. Physical Chemistry Chemical Physics. 2019
2. Narimoto T, Sakurai K, Okamoto A, Chatani E, Hoshino M, Hasegawa K, et al. Conformational stability of amyloid fibrils of beta2-microglobulin probed byguanidine-hydrochloride-induced unfolding. FEBS Lett. 2004;576(3):313–319.doi:10.1016/j.febslet.2004.09.024.
3. Baldwin AJ, Knowles TPJ, Tartaglia GG, Fitzpatrick AW, Devlin GL, Shammas SL, et al. Metastability of Native Proteins and the Phenomenon of Amyloid Formation. Journal of the American Chemical Society. 2011;133(36):14160–14163.doi:10.1021/ja2017703
4. Pedersen JS, Dikov D, Flink JL, Hjuler HA, Christiansen G, Otzen DE. The changing face of glucagon fibrillation: structural polymorphism and conformational imprinting. J Mol Biol. 2006;355(3):501–523.doi:10.1016/j.jmb.2005.09.100
