# Supplementary material for: The hydrophobic effect characterises the thermodynamic signature of amyloid fibril growth
Source: PLoS Comput Biol. 2020 May 4;16(5):e1007767. doi: 10.1371/journal.pcbi.1007767 (PMC7282669; doi:10.1371/journal.pcbi.1007767)
Supplement: S3 Methods — (PDF) [file pcbi.1007767.s003.pdf]

Meta analysis methods

Identification of the core regions of the amyloid fibrils

To be able to determine the hydrophobic contribution to the enthalpy of fibril elongation we measured or extracted from published data, we need to know the total hydrophobic surface area that contributes to fibril formation for each of the different fibrils.

To calculate the change in accessible hydrophobic surface area upon amyloid fibril elongation, we determined the fibril core-forming regions of each protein. PDB structures of the aggregating fibrils were available β-2-microglobulin (PDB ID:6GK3 [1]), α-synuclein (PDB ID:2N0A [2], residues 38-97) and for the microcrystals formed by the GNNQQNY peptide from Sup35 (PDB ID:2OMM [3]). In the case of glucagon, it has been reported that the fibril is formed by nearly the entire sequence of 29 amino acids [4]. For bovine α-lactalbumin, no high-resolution fibril structure is available to-date. However, α-lactalbumin is homologous to lysozyme [5], for which the aggregating region is known from limited proteolysis experiments [6]. Therefore, we queried both protein sequences using PSI-BLAST (accessed 25 January 2019) on the Swissprot database with default settings for two iterations. We selected the first twelve hits or the α-lactalbumin query (Uniprot: P00709, Q9N2G9, P00712, P00711, P09462, Q9TSN6, Q9TSR4, P00714, P61633, P30201, P61626, P00716) and added the lysozyme sequence and two hits of the lysozyme query with 70-80 percent sequence identity with lysozyme (Uniprot: P04421, P61631, P79811) to ensure that both proteins were represented in the sequence set. Subsequently, we performed multiple sequence alignment on these sequences using Clustal Omega with default settings. The resulting alignment was used to predict the aggregating region of α-lactalbumin from the known aggregating region of lysozyme. The estimated aggregating region was TFHT…GINY of α-lactalbumin (Uniprot P00711, region 48-122).

References

1. Iadanza MG, Silvers R, Boardman J, Smith HI, Karamanos TK, Debelouchina GT, et al. The structure of a β2-microglobulin fibril suggests a molecular basis for its amyloid polymorphism. Nature Communications. 2018;9(1):4517.doi:10.1038/s41467-018-06761-6.
2. Tuttle MD, Comellas G, Nieuwkoop AJ, Covell DJ, Berthold DA, Kloepper KD,et al. Solid-state NMR structure of a pathogenic fibril of full-length human α-synuclein. Nat Struct Mol Biol. 2016;23(5):409–415.
3. Sawaya MR, Sambashivan S, Nelson R, Ivanova MI, Sievers SA, Apostol MI, et al. Atomic structures of amyloid cross-β spines reveal varied steric zippers. Nature.2007;447(7143):453–457. doi:10.1038/nature05695.
4. Andersen CB, Hicks MR, Vetri V, Vandahl B, Rahbek-Nielsen H, Thogersen H,et al. Glucagon fibril polymorphism reflects differences in protofilament backbone structure. J Mol Biol. 2010;397(4):932–946. doi:10.1016/j.jmb.2010.02.012.
5. McKenzie HA, White FH. Lysozyme and alpha-lactalbumin: structure, function,and interrelationships. Advances in protein chemistry. 1991;41:173–315.
6. Frare E, Mossuto MF, de Laureto PP, Dumoulin M, Dobson CM, Fontana A.Identification of the core structure of lysozyme amyloid fibrils by proteolysis. JMol Biol. 2006;361(3):551–561. doi:10.1016/j.jmb.2006.06.055.
