# Supplementary material for: The hydrophobic effect characterises the thermodynamic signature of amyloid fibril growth
Source: PLoS Comput Biol. 2020 May 4;16(5):e1007767. doi: 10.1371/journal.pcbi.1007767 (PMC7282669; doi:10.1371/journal.pcbi.1007767)
Supplement: S1 Text — (PDF) [file pcbi.1007767.s004.pdf]

Supplementary data

Simulations with model parameters affecting the enthalpic and entropic contributions to fibril stability

We also investigated how the cold denaturation and destabilisation depend on the different model parameters. In our model we can adjust the stability of the fibril by changing the strength of the hydrogen bonds (S2 Fig). The stability obtained through H-bonds is purely enthalpic in our model. Similarly, we can change the entropic contribution to fibril stability by changing the β-strand propensity (S3 Fig); this term is purely entropic in our model. The simulations show that the stability of aggregates at low temperatures depends on the stability at physiological temperatures, see S2 Fig and S3B Fig.

Raw ITC data

S4 Fig shows the raw data from some of the ITC experiments used to produce the plots in Fig 5 of the main manuscript. The details about the injection volumes and monomer concentrations can be found in the Fig caption. In order to analyse these data, a baseline was defined and each of the peaks due to the injection of monomer was integrated numerically. In addition, we have also acquired atomic force microscopy images (S5 Fig), which illustrated both the preparation of the seed fibrils of α-synuclein through sonication, which increases the seeding efficiency [1], as well as the subsequent growth during the ITC experiment upon injection of soluble protein.

Fibril stability in the presence of denaturants

Our model predicts that cold denaturation of amyloid fibrils is only observed for fibrillar systems that are not very highly thermodynamically stable with respect to the soluble state of the protein. This condition arises from the requirement that the low temperature-induced weakening of the hydrophobic effect leads to a sufficient overall loss in stability for the equilibrium concentration of soluble protein to increase substantially.

Published data show that α-synuclein amyloid fibrils are among the least stable amyloid fibrils characterised to-date, in agreement with the observation that these fibrils are among the few that have been found to display cold denaturation. We have performed additional experiments on the thermodynamic stabilities of glucagon and α-lactalbumin amyloid fibrils under the same conditions under which we have performed the ITC experiments (S6 Fig).

References

1. Buell AK, Galvagnion C, Gaspar R, Sparr E, Vendruscolo M, Knowles TPJ, et al. Solution conditions determine the relative importance of nucleation and growth processes inα-synuclein aggregation. Proc Natl Acad Sci U S A.2014;111(21):7671–7676. doi:10.1073/pnas.1315346111
