# Supplementary figures and images for: The hydrophobic effect characterises the thermodynamic signature of amyloid fibril growth
Source: PLoS Comput Biol. 2020 May 4;16(5):e1007767. doi: 10.1371/journal.pcbi.1007767 (PMC7282669; doi:10.1371/journal.pcbi.1007767)

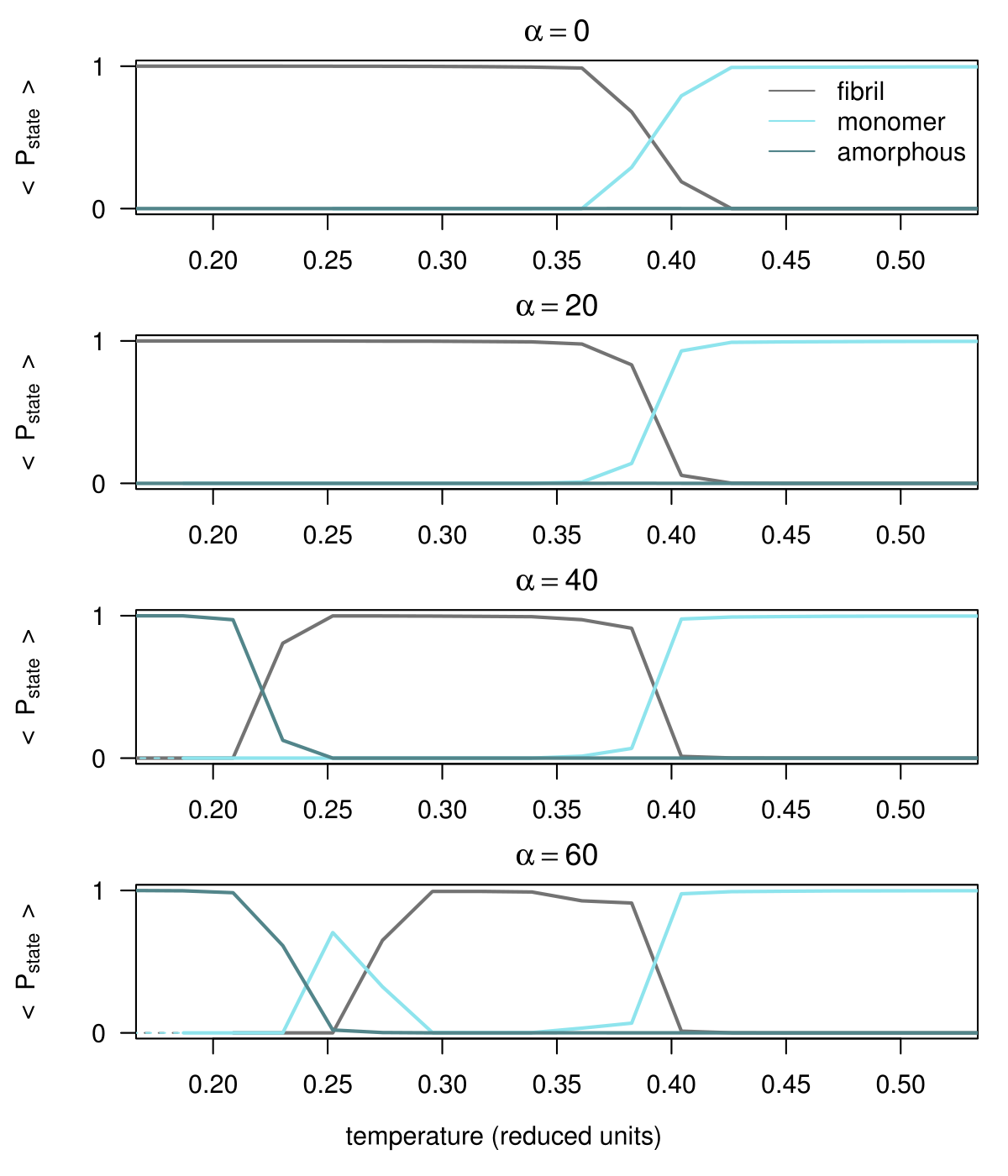

Supplement: S1 Fig — Here, we explored the effect of the strength of the hydrophobic temperature dependence, α, on the stability of the aggregates. Three different states can be discerned: the fully formed fibril (black), denaturation of the fibril into monomers (cyan) and an amorphous aggregate where the two additional layers are not fully formed. Only in the models with a hydrophobic temperature dependence, cold destabilisation (α = 40, 60), or cold denaturation into monomers, may be observed (α = 60). The dashed lines indicate that the state has not been observed (sampled) in the simulations at the corresponding temperature. Note that the reduced temperature units for this model can be interpreted to have a freezing point around T = 0.18, and boiling point just above T = 0.4. (TIF) [file pcbi.1007767.s007.tif]

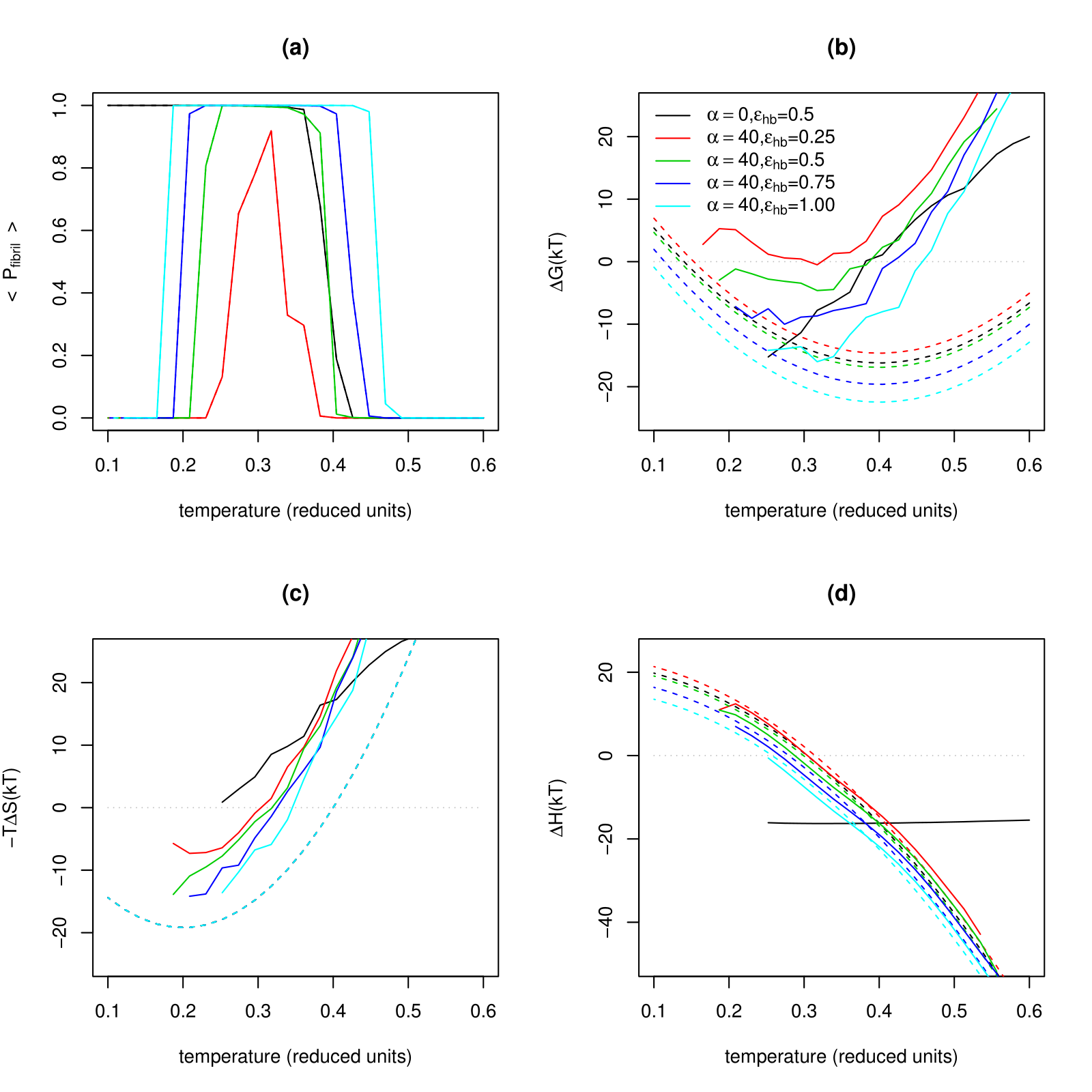

Supplement: S2 Fig — We explored the stability of the fibrillar state for different values of the hydrogen bond strength (ϵhb) in the model. For varying values ϵhb, and α = 40 the state diagram for the fibrillar state (A), the free energy (B), and corresponding entropic (C) and enthalpic (D) contributions are shown. Increasing the hydrogen bond strength makes the fibril more stable (b), resulting in a wider temperature range over which the fibrillar state is stable (a). Dotted lines indicate estimates for the hydrophobic contributions showing ΔG^hydr, -TΔS^hydr and ΔE^hydr; these estimates are generated using Eqns. 13, 15 and 14 with corresponding α, ΔCh = −6 and with an offset, Eint = ΔH based on simulations with the equivalent peptide for α = 0. (TIF) [file pcbi.1007767.s008.tif]

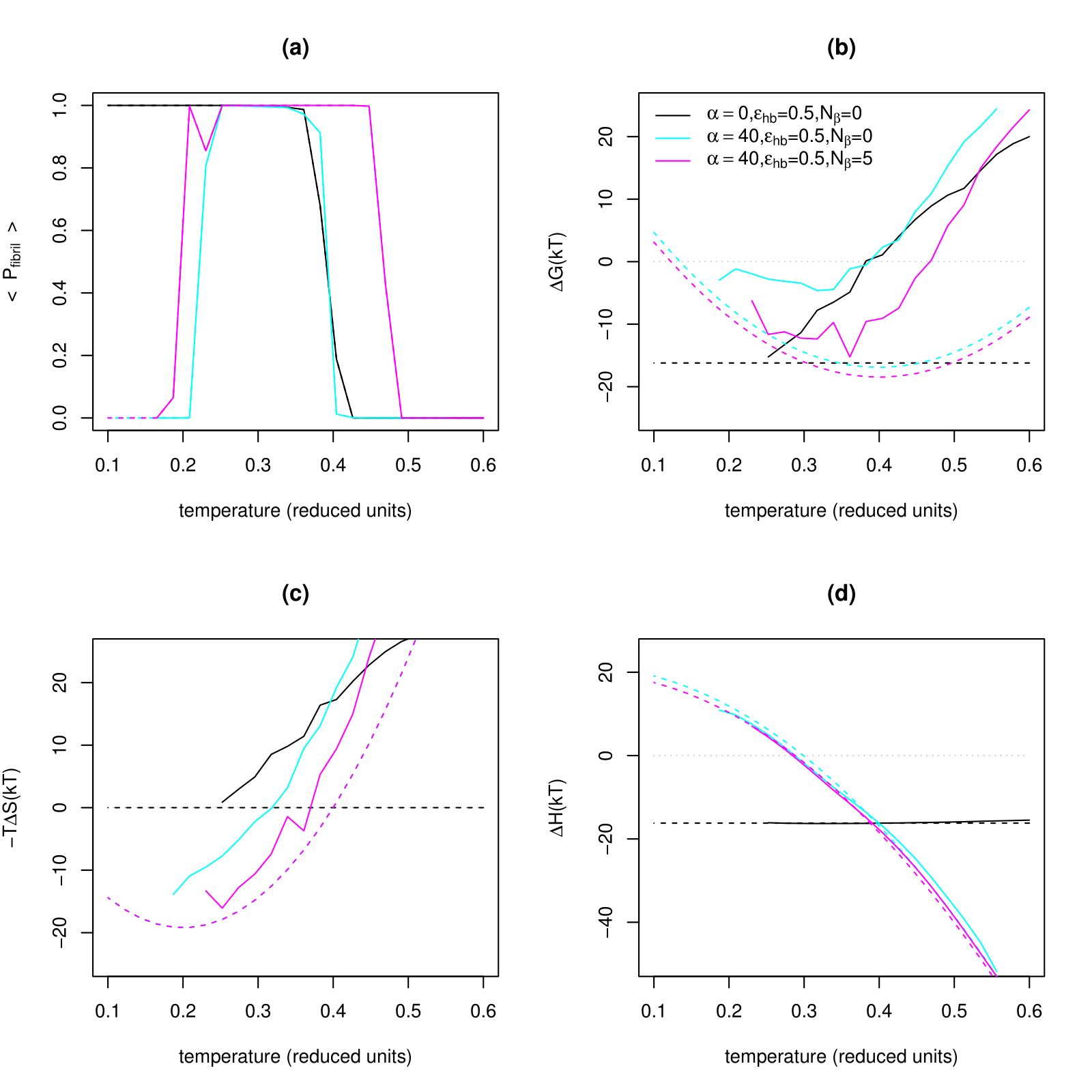

Supplement: S3 Fig — We explored the stability of the fibrillar state for different values of the (entropic) propensity of β-strand state (Nβ) in the model. For varying values Nβ, and α = 40 the state diagram for the fibrillar state (A), the free energy (B), and corresponding entropic (C) and enthalpic (D) contributions are shown. Increasing the β-strand propensity makes the fibril more stable (B), resulting in a wider temperature range over which the fibrillar state is stable (A). Dotted lines indicate estimates for the hydrophobic contributions showing ΔG^hydr, -TΔS^hydr and ΔE^hydr; these estimates are generated using Eqns. 13, 15 and 14 with corresponding α, ΔCh = −6 and with an offset, Eint = ΔH based on simulations with the equivalent peptide for α = 0. (TIF) [file pcbi.1007767.s009.tif]

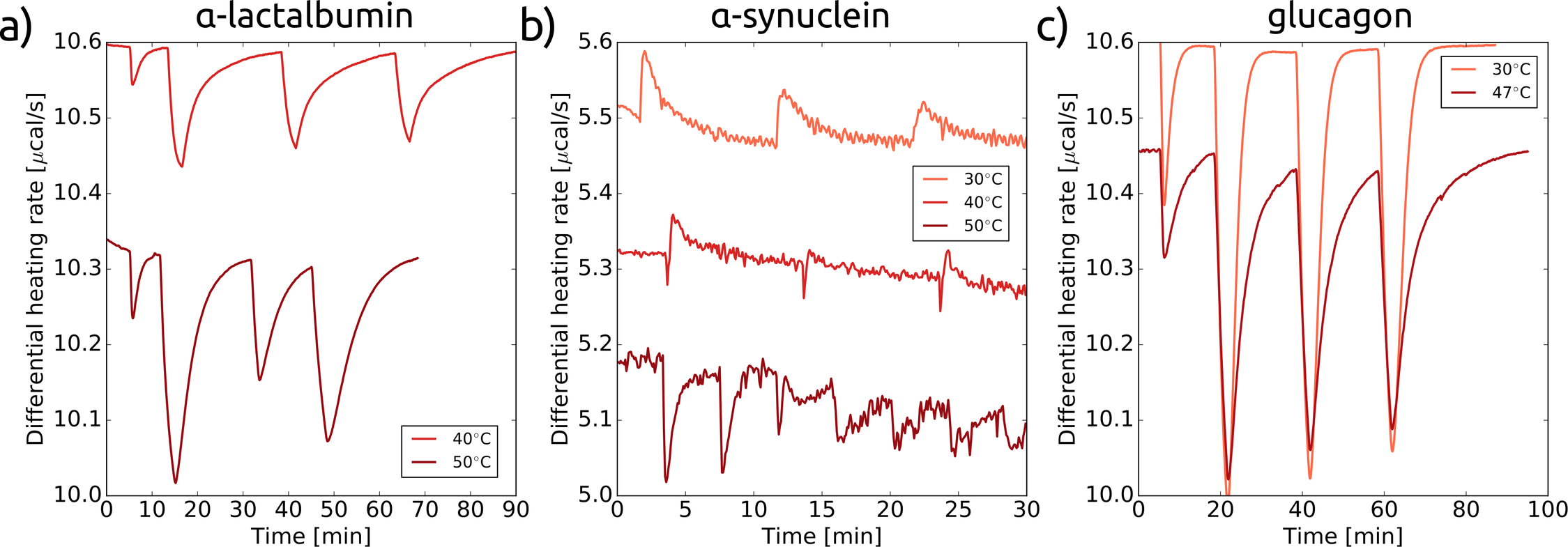

Supplement: S4 Fig — Raw data of ITC experiments are shown for experiments where monomer solutions were titrated into seed fibril suspensions. Experiments were performed with a VP-ITC (A,C) and an ITC200 (C) instruments. (A) Injections of 10, 80, 80, 80μl (40°) and 10, 80, 40, 80μl (50°) of a solution of α-lactalbumin (50 μM in 10 mM HCl+100mM NaCl) into a suspension of sonicated seed fibrils. (B) Injections of 2 μl of solutions of monomeric α-synuclein at 380 μM (50°C), 390 μM (30°C) and 430 μM (40°C) into seed fibril suspensions. (C) Injections of 20, 80, 80, 80μl (30 and 47°) of a solution of glucagon (100 μM in 10 mM HCl+30mM NaCl) into a suspension of sonicated seed fibrils. (TIF) [file pcbi.1007767.s010.tif]

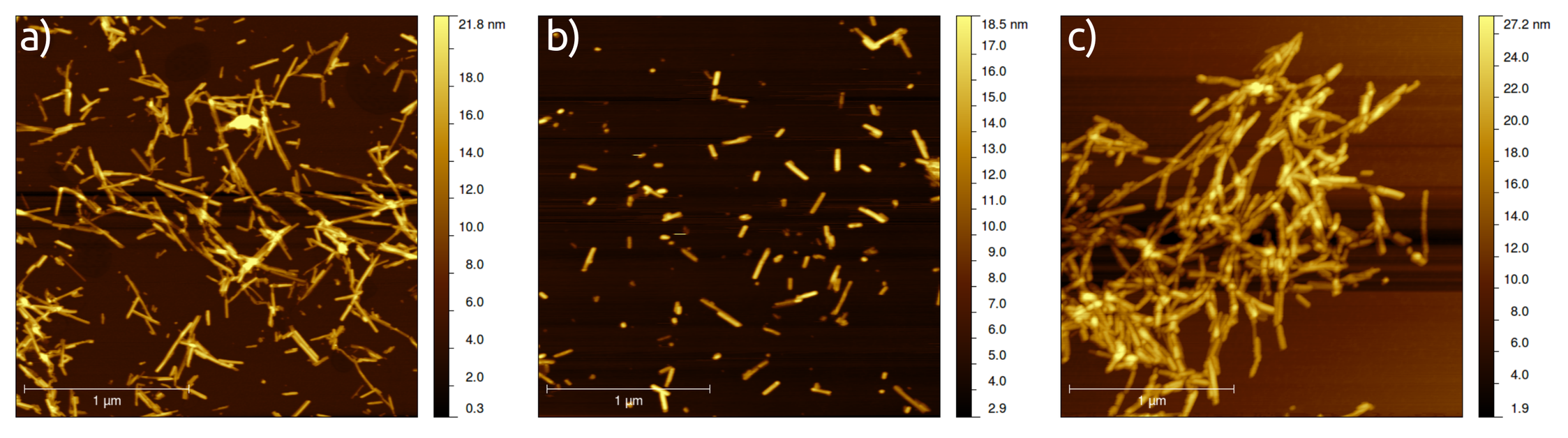

Supplement: S5 Fig — Atomic force microscopy (AFM) images were taken of seed fibrils before sonication to shorten the length distribution and enhance the seeding efficiency (A), after 10 s of sonication with a sonication probe (B) and after an ITC experiment (C), where the fibrils (40 μM) had been incubated with a total of 60 μM of monomeric α-synuclein. (TIF) [file pcbi.1007767.s011.tif]

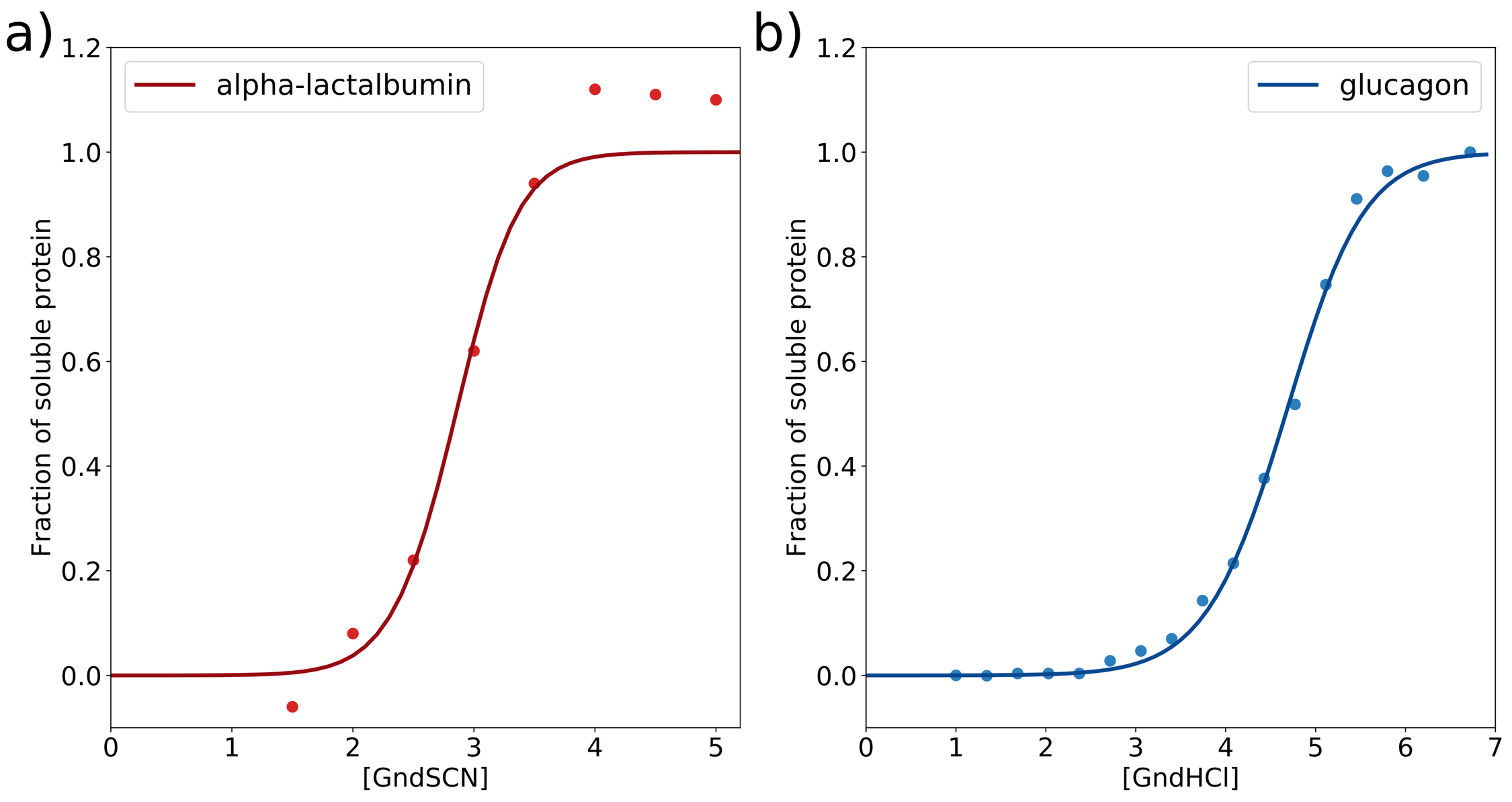

Supplement: S6 Fig — (A) α-lactalbumin amyloid fibrils depolymerised with the strong denaturant GndSCN. (B) glucagon amyloid fibrils depolymerised with GndHCl. The values of the free energy difference between the soluble and fibrillar states are -52.5 kJ/mol (α-lactalbumin) and -51.2 kJ/mol (glucagon). These values should be compared with the one determined for the considerably less stable α-synuclein amyloid fibrils of -33.0 kJ/mol [36] or -37.4kJ/mol (this study) in PBS buffer. (TIF) [file pcbi.1007767.s012.tif]

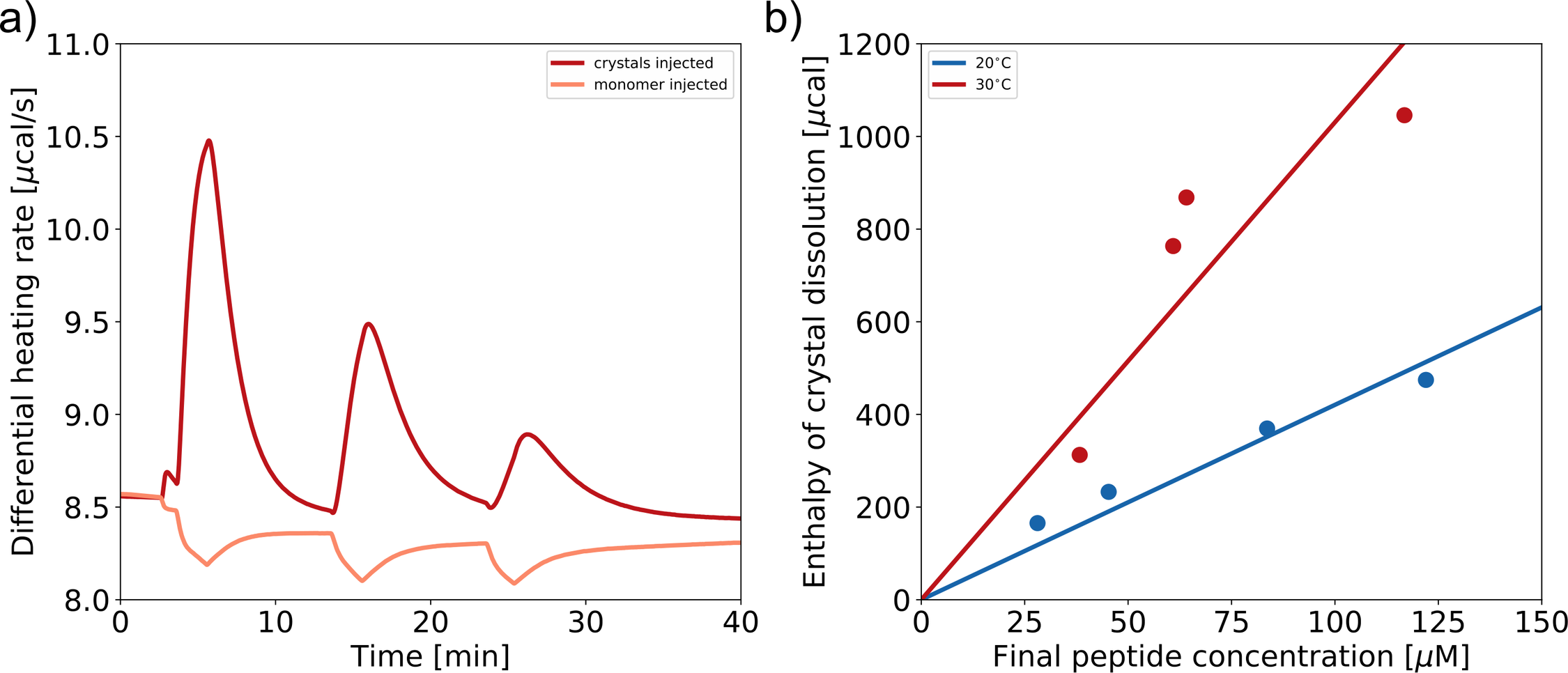

Supplement: S7 Fig — (A) Raw ITC data of the injectionof GNNQQNY crystals and monomer into pure water. Experimental details see the Materials and methods section. (B) Summary of the calorimetric results of GNNQQNY crystal dissolution. The data points are corrected for the exothermic heats of dilution of the monomeric content of each injection. The linear fits to the data sets at the two temperatures are used to determine the molar enthalpies of crystal dissolution, which corresponds to the negative of the molar heats of crystal growth. (TIF) [file pcbi.1007767.s013.tif]

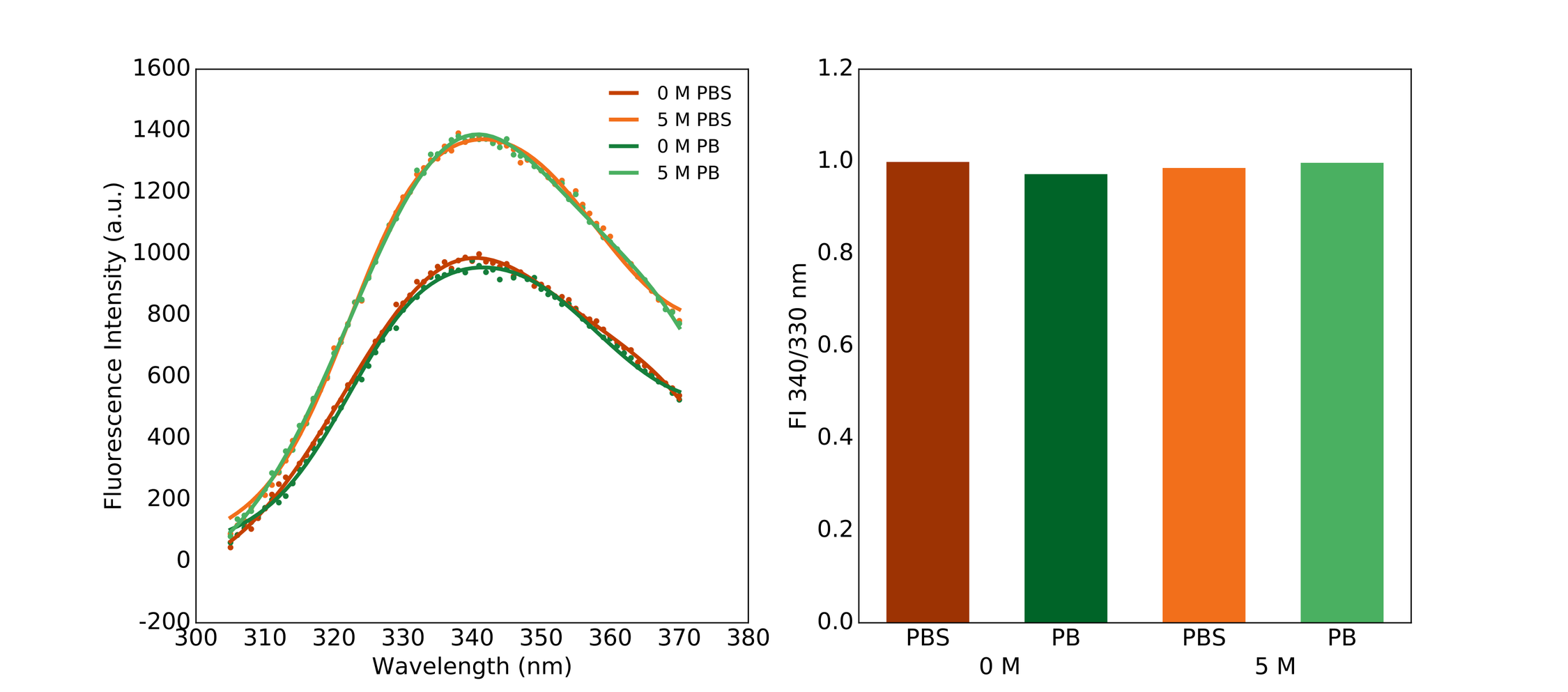

Supplement: S8 Fig — Left: Intrinsic fluorescence of monomeric F94W α-synuclein was measured in PB pH 7.4 in the presence and absence of 150 mM NaCl and in the presence and absence of 5 M urea. Right: The fluorescence intensity ratios at 340 and 330 nm were plotted for all 4 conditions, confirming that this ratio is nearly constant for monomer under all the observed conditions. These results provide strong support for our interpretation of the change in fluorescence intensity ratio with increasing urea concentration as reflecting fibril depolymerization. (TIF) [file pcbi.1007767.s014.tif]

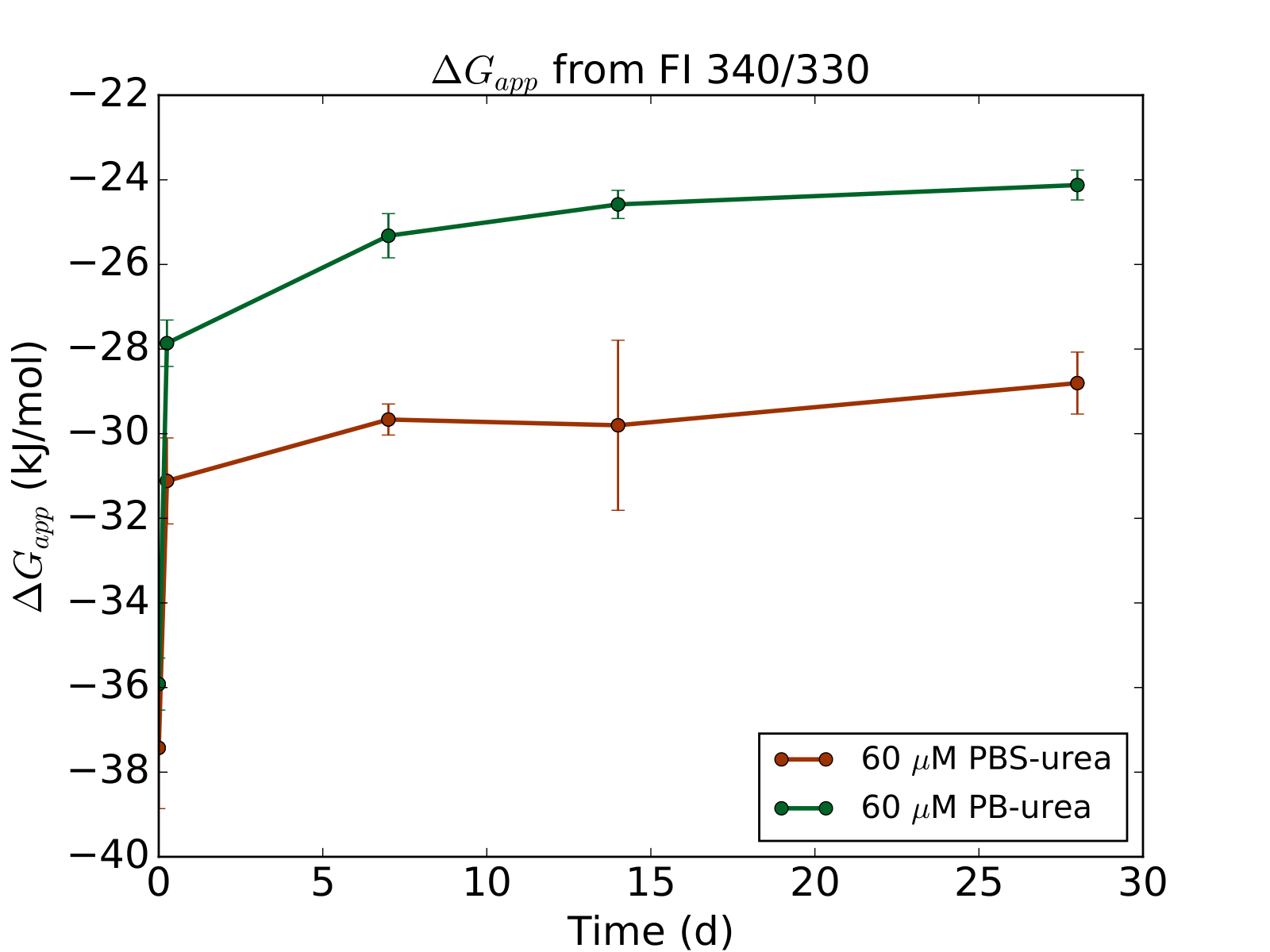

Supplement: S9 Fig — Urea denaturation series of F94W α-synuclein fibrils in the presence and absence of 150 mM NaCl were incubated at 4°C for 4 weeks and the intrinsic fluorescence spectra were measured and the overall denaturation curves determined and analysed with the isodesmic polymerisation model. The determined free energy values were plotted as a function of equilibration time. It can be seen that equilibrium is reached after a few days. (TIF) [file pcbi.1007767.s015.tif]

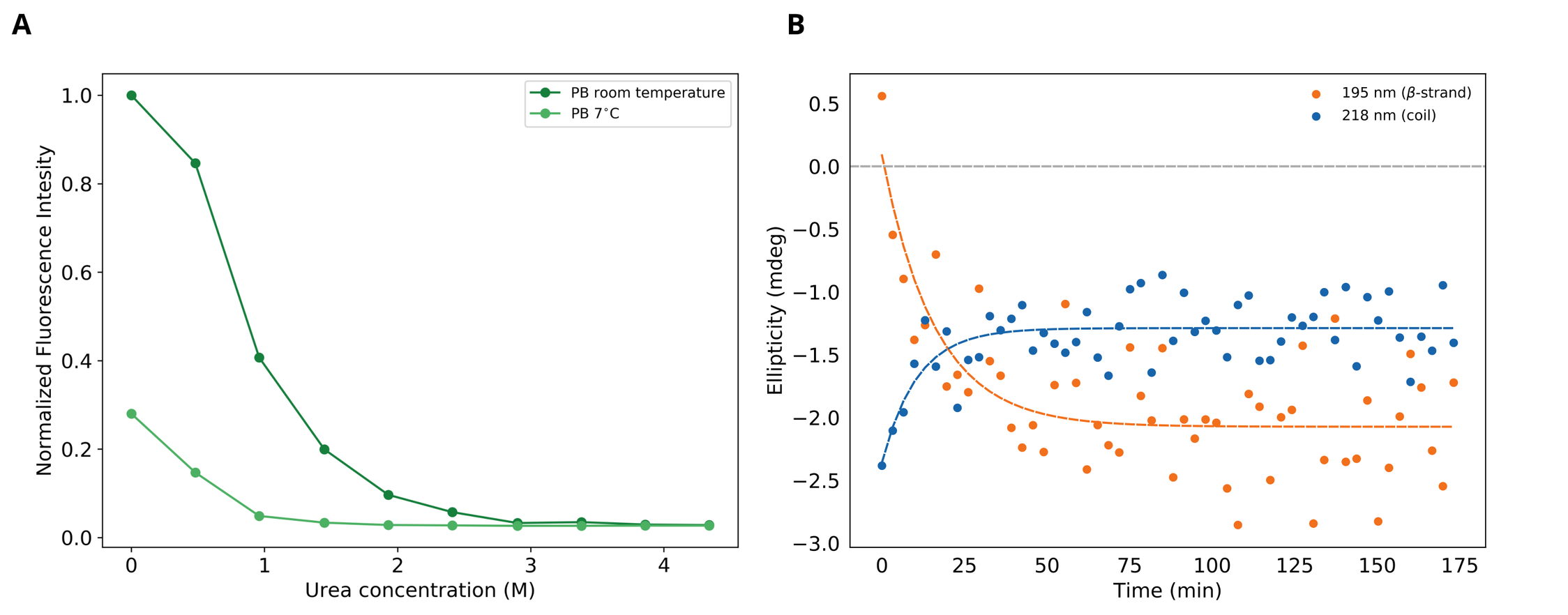

Supplement: S10 Fig — (A) α-synuclein amyloid fibril stability as a function of urea concentration at room temperature and after equilibration at 7°C, using Thioflavin-T fluorescence as a read-out for the degree of fibril depolymerisation. (B) Time course of partial fibril depolymerisation at 7°C, as followed by circular dichroism (CD) spectroscopy. See the Materials and methods section for experimental details. The data have been fitted to negative exponential functions, corresponding to the expected behaviour of fibril dissolution, whereby the number of fibrils is approximately constant during most of the dissociation time course and where the rate of dissociation is proportional to the difference between the equilibrium concentration and the actual concentration of monomer. (TIF) [file pcbi.1007767.s016.tif]
