# Supplementary material for: The hydrophobic effect characterises the thermodynamic signature of amyloid fibril growth
Source: PLoS Comput Biol. 2020 May 4;16(5):e1007767. doi: 10.1371/journal.pcbi.1007767 (PMC7282669; doi:10.1371/journal.pcbi.1007767)
Supplement: Attachment — Submitted filename: ResponsToReviewers_Abeln.pdf [file pcbi.1007767.s017.pdf]

Dear dr. Nikolay V. Dokholyan and prof. Nir Ben-Tal,

We are grateful to the editors and reviewers for taking the time to read our manuscript, and are pleased that they deem this work of sufficient quality to be published in PLoS Computational Biology. The reviewers appreciated the combined use of a coarse-grained model and *in vitro* experiments to study amyloid fibril elongation, a process with important medical implications.

We appreciate the suggestions provided by the reviewers to further improve the quality of the manuscript, and have made the following changes in response to the reviewers' comments (line and page numbers correspond to the clean manuscript without tracked changes):

Reviewer #1:

1. Comment: *"For the CG model, T0 value (the temperature-dependent terms of protein-solvent interaction) was not given."*

   Response: We have added that T0=0.4 (in relative units) for the GC model to the Materials and Methods section of the manuscript (page 7 line 187).

2. Comment*: "For the depolymerisation experiments and cold-denaturation of fibrils (e.g., Fig. 6), it would be great if additional approaches could be used to cross-validate the cold-denaturation, such as AFM or TEM imaging."*

   Response: We have recently published a study (Vettore et al., Phys Chem Chem Phys 2019), in which we characterize in detail the use of chemical depolymerisation for the study of the thermodynamic stability of amyloid fibrils, and we have made the reference to this study more explicit (section "Fibril stability in the presence of denaturants", page 9 line 269). The use of high denaturant concentrations in such experiments renders imaging methods somewhat challenging, because of the residual denaturant often present in significant concentrations. Therefore, we performed additional spectroscopic experiments, this time with the amyloid reporter dye Thioflavin-T, and these experiments confirmed the dependence of alpha-synuclein amyloid fibril stability on both temperature and salt concentration that we had found from in our intrinsic fluorescence experiments with the Trp variant of alpha-synuclein. We have added this data as a supplementary figure (Supplementary figure S10, page 34).

Reviewer #2 mainly expressed his interest in this work and had no comments that need to be addressed in the manuscript.

We hope that the reviewer comments have been sufficiently addressed for the manuscript to be accepted into PLoS Computational Biology.

Yours sincerely,

Alexander Buell and Sanne Abeln, on behalf of all coauthors
